# Maternal separation blunted spatial memory formation independent of peripheral and hippocampal insulin content in young adult male rats

**DOI:** 10.1371/journal.pone.0204731

**Published:** 2018-10-17

**Authors:** Soheila Maghami, Homeira Zardooz, Fariba Khodagholi, Fatemeh Binayi, Roya Ranjbar Saber, Mehdi Hedayati, Hedayat Sahraei, Mohammad Ali Ansari

**Affiliations:** 1 Neuroscience Research Center, Shahid Beheshti University of Medical Sciences, Tehran, Iran; 2 Department of Physiology, School of Medicine, Shahid Beheshti University of Medical Sciences, Tehran, Iran; 3 Neurophysiology Research Center, Shahid Beheshti University of Medical Sciences, Tehran, Iran; 4 Neurobiology Research Center, Shahid Beheshti University of Medical Sciences, Tehran, Iran; 5 Cellular and Molecular Research Center, Research Institute for Endocrine Sciences, Shahid Beheshti University of Medical Sciences, Tehran, Iran; 6 Neuroscience Research Center, Baqiyatallah University of Medical Sciences, Tehran, Iran; 7 Laser and Plasma Research Institute, Shahid Beheshti University, Tehran, Iran; Technion Israel Institute of Technology, ISRAEL

## Abstract

This study explores the effects of maternal separation as a chronic early life stress (ELS) on pancreatic islets insulin content and secretion, and their potential relationship with the hippocampus insulin content and spatial memory in young adulthood.

Male rat offspring were divided into two groups: stress (STR) and non-stress (non-STR) groups. The animals of the STR group were separated from their mothers during postnatal days (PND) 1 to 21. During the weaning time, that is, PND-0 to PND-21, the body weight and length of the pups were measured. Blood samples were collected on PND-1, 21, 29 and 34 and during young adulthood (53±2 days) to determine plasma corticosterone and insulin levels. The young adult animals were also tested for spatial memory. One day after the memory test, the animals were decapitated and their pancreases were removed to measure the islets insulin content and secretion. Finally, the animals’ hippocampi were isolated to determine their insulin content and insulin receptor protein amounts.

During the period of weaning, the body weight and length of pups belonging to the STR group were significantly lower as compared to those in the non-STR group. Maternal separation did not change the plasma levels of insulin but increased plasma corticosterone levels from PND-21 to young adulthood and also reduced the islets insulin content but did not affect insulin secretion and the hippocampus insulin content and insulin receptor protein amount. Although, at the end of the memory tests, rats of the STR group reached the escape box at almost the same time and distance and with the same errors as rats of the non-STR group, the distance traveled to reach the escape box showed a steep reduction in the non-STR group as compared to the STR group after the first trial. Moreover, as compared to the STR group, the non-STR group showed an increasing trend for direct strategy to find the escape box. The islets insulin content and secretion, and the plasma insulin concentration were not significantly correlated with the hippocampus insulin content.

From the results of the present study, it appears that the main behavioral effect of the maternal separation stress in the spatial memory task was to impair the strategy used by the animals to reach the escape box. This may indicate that maternal separation stress affects brain regions other than the hippocampus. Moreover, due to the reduction of the body weight and length of offspring belonging to the STR group, it should be further considered that both maternal separation and early life malnutrition are directly (and mechanistically) linked to cognitive alterations later in life in ways that are not dependent on peripheral and hippocampal insulin content.

## Introduction

Evidence suggests that chronic early life stress (ELS) has harmful effects on cognitive functions such as learning and memory in humans [1] and animals in later life [2, 3]. Studies have shown that the harmful effect of chronic stress on memory is more prominent when the stress is applied during infancy [4, 5]. The justification is that when stress is applied during critical periods of brain development (that is, in childhood and youth), it will have long-term disruptive effects on the brain structure and the individual’s behavior [6]. In support of this, Sousa et al. reported that maternal separation stress during infancy, from postnatal day (PND)-2 to PND-14, reduces spatial memory formation in male rats 26 days after the end of the stress period and when the rats are between 40 and 70 weeks old. This finding shows that maternal separation stress has a long-term detrimental effect on spatial memory in male rats [5]. According to researchers, one of the main factors causing the long-term damaging effects of ELS on spatial memory is the persistent effects that stress can have on the neuronal structure and function of the hippocampus, which is involved in memory processing [7]. These damaging effects might be partly due to malnutrition of the pups because the maternal care they receive changes after separation from the dams [8, 9].

Another view is that insulin has neuroprotective and neurotropic effects [10] on the central nervous system, which improves cognitive functions, especially memory processing [11]. The memory-enhancing effect of insulin is exerted via its receptors in the brain, which are predominantly expressed in the hippocampal CA1 region [12], one of the main regions involved in spatial memory [3, 13]. Zhao et al. [13] also showed that insulin receptors are involved in the process of memory formation, possibly by regulating synaptic activities (such as neuronal transmission and synaptic facilitation). The results of different human and animal studies suggest that insulin can improve spatial memory. For example, the application of intranasal insulin in Alzheimer’s patients improved their spatial memory [14]. The intra-cerebro ventricular (ICV) and direct injections of insulin into the hippocampus in a rat model with Alzheimer's disease were also found to improve spatial memory [12]. On the other hand, a positive linear relationship was observed between serum levels of insulin and the brain insulin content [4]. For example, Magarinos et al. [15] observed that the lack of insulin secretion and decreased plasma insulin causes memory loss in animal models with diabetes. Previous studies have also shown that different kinds of stress in adulthood can cause dysfunction of the pancreatic beta cells and reduce the islets insulin secretion and content [16, 17]. Plasma insulin concentrations reduced significantly in adult Wistar rats separated from their mothers on PND 2–21 (3 h/day) as compared to the control group [4]. Stress may affect insulin receptors’ expression in the brain. Osmanovic et al. [18] showed that, brain insulin receptor gene expression decreases in adult rats after a chronic increase in exogenous corticosterone. Moosavi et al. [12] studied male adult rats and demonstrated the protective effects of insulin against the destructive effects of restraint stress on spatial memory. According to the findings of these investigations, it could be suggested that the changes in pancreatic islets insulin secretion and plasma insulin concentration in rats exposed to ELS (in the form of maternal separation) may be related to the hippocampus insulin content on one hand, and the possibility of spatial memory impairment on the other. However, this reasoning needs further demonstration.

The present study was conducted to explore the effects of chronic ELS in rats as a result of maternal separation on first, the animals’ spatial memory, which is considered as a main function of the hippocampus, and second, the pancreatic isolated islets insulin secretion and content, the plasma insulin concentration and the hippocampus insulin content. The amount of insulin receptor protein was also measured in the hippocampus.

## Materials and methods

### Animals

Female (200±10 g) and male (250±20 g) Wistar rats purchased from Pasture Institute in Tehran, were mated overnight and separated at 9 am. After detecting sperm in their vaginal smear, the pregnant rats were kept until delivery. Twenty litters were used in this study. The litters were culled to 6–7 pups. The male pups of each litter were randomly allocated to the stress (STR) and non-stress (non-STR) groups (n = 34/group) on the day they were delivered (PND-0). The animals of each group came from different litters. Only male pups were examined in this study due to the fact that the existing knowledge on the mechanisms of maternal separation stress is still incomplete and because the female sex hormones have complex effects on the brain [19, 20] and may interact with the stress hormones; however, the findings can act as a basis for future studies on female rats. The male pups were thus kept in a temperature-controlled room (22±2°C) with a 12-h light/dark cycle (lights turned on at 7 am). Standard rat chow (Pars Animal Feed Co., Tehran, Iran) and tap water were made available to the rats throughout the entire experiment. At young adulthood (53±2 days) [21], the animals were tested in each group for their spatial memory for four days using a Barnes maze; the tests were conducted between 9 am and 4 pm. The day after the last memory trial, the animals were decapitated and their trunk blood samples were collected and their pancreases removed for islet isolation and determination of their insulin content and secretion. Finally, their brain was removed and their hippocampus isolated to measure their insulin content and insulin receptor protein amounts. The Animal Care and Use Committee of Shahid Beheshti University of Medical Sciences (IR.SBMU.SM.REC.1394.149) approved all the procedures.

### Maternal separation protocol

In order to induce maternal separation stress, the animals in the STR group were separated from their mothers from PND-1 to PND-21 and placed in a clean cage similar to their home cage, and transported to a room with controlled temperature (30–32°C) for three hours (from 9 am to 12 am) [4]. The pups were then carefully returned to their home cage. The non-STR group animals remained with their mothers, who were not the same as the mothers in the STR group.

### Maternal behavior assessment

#### Pup retrieval test

Some maternal behaviors were evaluated according to the experiments described by Aguggia et al. [9] with minor modifications. After postpartum, on PND-3, the pup retrieval test was performed to evaluate certain behavioral parameters (latency to retrieve the pups back to the nest and the number of pups successfully retrieved, latency to first pup contact and frequencies of rearing and grooming) in the dams of both the non-STR and STR pups, which are called the non-STR dams and the STR dams. For this purpose, the non-STR dams were removed from their home cage for 3 min. During this time, the pups were translocated to the opposite side of the nest. This procedure was performed for the STR dams 3 min before the end of the pup separation as well. The time spent by the dams to return the pups to the nest and the number of pups successfully returned were then calculated. The latency to sniff or contact the first pup as well as the dams’ rearing and grooming frequencies were evaluated during the test, which lasted for 10 min (the cut-off time). In a situation where the dams did not return the pups during the cut-off time, the pups were returned to the nest by the experimenter.

### The anxiety-like behavior test for the dams

After weaning on postpartum day 22, between 8 am and 9 am, the dams’ (6/group) anxiety-like behavior was assessed using the elevated plus maze (EPM) apparatus [22]. The apparatus consisted of four crossed arms made from black Plexiglas. The arms were 100 cm long and 10 cm wide. Two arms had no walls (open arms) and two had a 50×50-cm wall (closed arms). The arms were separated by a central square platform (10 x 10 cm). Each EPM experiment session lasted for 5 min. The test was initiated by placing the animal on the central platform of the maze while facing one of the closed arms. The dams’ position and movement were recorded and analyzed online using EthoVision (Noldus, USA). Four parameters were recorded in the test: The time spent by the dams in the open arms and also in the closed arms, the total distance traveled by the dams during the test session and the rats’ velocity in the maze. For each animal, the percentage of open arms time (OAT%) was calculated as follows:

The percentage of time spent in the open arms = (Time spent in the open arms / Time spent in the open arms + Time spent in the closed arms) ×100

A significant reduction in OAT% indicates an anxious-like behavior in this test.

### The forced swimming test

The forced swimming test was done to evaluate the degree of depression in the dams (6/group) on postpartum day 22. A glass cylinder, 50 cm in height and 20 cm in diameter, was filled with 23±1°C tap water in proportion to the rat’s size (such that its forelimbs did not touch the bottom of the container). The rats were gently released into the water from 20 cm above. The experiment was carried out for all the animals between 9 am and 12 am. There were two sessions, the first is the pretest and lasted for 15 min, and the second which is the main test, was carried out 24 h later (on postpartum day 23), and lasted for 5 min. The animals’ behavior was recorded by a camera. The immobility frequency was measured during the 5 min of the main test as an indicator of depression [23].

### The body weight and length of the pups during the weaning period

From the day of birth (PND-0) to the end of the weaning time (PND-21), the pups’ body weight (using a digital scale with a precision of 0.1 g) and length (head to tail length + tail length, using a Vernier caliper with a precision of 0.1 mm) were measured.

### Spatial memory test

The spatial memory tests were performed using a Barnes maze [24]. The maze consisted of a black circular Plexiglas platform (95 cm in diameter) with 12 holes (10 cm in diameter) placed at the edge of the platform with equal spacing. The platform distance from the ground was 140 cm in order to prevent the rats from skipping the platform to the ground [25]. A black Plexiglas box (escape box) was placed under one of the holes (the target hole). This escape box had the same position for each animal throughout the test; however, the platform itself was rotated to confuse the animals’ sense of smell and to prevent them from following distinct smells. After each trial, the platform and the escape box were cleaned with ethanol 20%. Color papers with different shapes were put up on the walls in the test room and other devices in this room were kept fixed and could be used as cues by the rats to find the escape box [25]. The animals’ movement on the maze was recorded by a CCTV camera located 85 cm above the maze. An EthoVision (Noldus, USA) was used to monitor and record the animals’ positions, the distance the animals had moved and the length of time they had spent ambulating, resting and moving but not ambulating (twitching and making circular movements) [25].

The memory tests were performed in a room that was sound-attenuated over four consecutive days (with four trials per day). At young adulthood (PND-53±2), eight animals were tested in the maze per group (8 litters per group), and while the animals were being tested, the others were kept in their home cage in the animal room. One day before starting the memory tests, the animals were habituated to the maze environment. They were put in the escape box for 2 min, then placed directly in the target hole and allowed to enter and stay in the escape box, beneath the hole, for 2 min [25]. On day 1 of the memory test, after giving the animal 15 min to be habituated to the test environment, at 9–9:30 am, the animals were put in the center of the platform under a black bucket. Two 30-W LED light bulbs in the ceiling were then turned on and the bucket was lifted. The LED light had a visible spectrum range of about 440 to 700 nm with a peak at lambda = 590 nm (S1 Fig) and the intensity of the light was 764 candela at the maze level. From this time, the animals were monitored until they reached the target hole and entered the escape box. The cut-off time for each animal was a maximum of 180 s in the maze in each trial. Errors (indicated by the animal moving at the edge of a hole other than the target hole) and the distance and time spent to get into the escape box were measured during each trial [25]. The strategy used by the animals to reach the escape box was assessed. They used three types of strategies (direct, serial and random) to reach the escape box. A direct strategy meant that the animal was moving directly from the center of the maze to the escape box. A serial strategy meant that the animal was moving serially between the holes until it reached the escape box. Lastly, a random strategy meant that the animal was moving randomly in the center of the maze until it reached the escape box [26]. When the animal entered the escape box, it was allowed to stay for 2 min, and then removed to its cage for 15 min. Another trial was then begun, and this procedure was repeated four times per day [25].

### Blood sampling

One day after performing the behavioral tests, blood samples were taken from the dams (6/group) after 16 h of fasting by decapitation under isoflurane anesthesia (6.5 ml/l/kg of isoflurane/desiccator volume/rat body weight) (Baxter, USA) [27] to determine the plasma corticosterone level.

To determine the pups’ blood corticosterone level, the pups in each group (10 litters per group) were anesthetized with isoflurane on PND-1 and decapitated before the stress session, and their trunk blood was collected for determination of their blood corticosterone level. This group was taken as the pre-stress-exposure group (before). After the first exposure to stress on PND-1, ten pups of each group (10 litters per group) were also anesthetized and decapitated on day 1 of the stress session and their trunk blood was collected for determination of their blood corticosterone level. On PND-21, after the last stress session, and also on PND-29, 36 and 43, blood samples were obtained from the rats’ retro-orbital sinus (10 rats/group, 10 litters/group) while they were under isoflurane anesthesia in order to determine their plasma corticosterone level. One day after the end of the memory tests (PND-53±2), the young adult animals in both groups (14 rats/group, 14 litters/group) were anesthetized and decapitated at 8–8.30 am after 16 h of fasting and their trunk blood was collected into an Eppendorf tube containing 5 μl of heparin (5000 IU/ml, Caspian Tamin, Rasht, Iran) per 1 ml of blood. The tubes were then centrifuged at 503 ×g and 4°C for 10 min and their plasma was separated and kept at -70°C for measurement of the corticosterone (10 rats/group) and insulin (14 rats/group) levels.

#### Plasma insulin and corticosterone level assessment

Plasma insulin and corticosterone levels were measured using a rat insulin ELISA kit (minimum detection: 0.07 μg/l; Mercodia, Sweden) and a rat corticosterone ELISA kit (minimum detection: 25 ng/ml; ZellBio, Germany). The measurements were performed in one run and the intra-assay coefficients of variation for the insulin and corticosterone measurements were 3.2 and 6.1%. Plasma corticosterone changes were assessed as a percentage of the pre-stress-exposure value of corticosterone.

### The islet isolation procedure

Islet isolation (6 rats/group, 6 litters/group) was performed using the collagenase technique proposed by Lacy and Kostianovsky (1967) with slight modifications [28]. The entrance of the common bile duct to the duodenum was clamped, the duct was cannulated with a polyethylene catheter (Portex Intravenous Cannula 2.5 F, 0.75 mm OD) and 10 ml of cold Hank’s buffer [containing in mM: NaCl, 137; KCl, 5.4; CaCl_2_, 1.2; MgSO_4_ 7H_2_O, 0.8; Na_2_HPO_4_ 2H_2_O, 0.3; KH_2_PO_4_, 0.4; NaHCO_3_, 4.2 (Merck, Germany)] [17], in which collagenase P (Roche, Cat. #11 213 865 001, Germany, 0.44 mg/ml) was diluted, and gently injected into the duct. The inflated pancreas was removed and placed into a Petri dish and cleaned of non-pancreatic tissue. The pancreas was then placed into a 50-ml falcon tube and incubated in a 37°C water bath for 17 min. By adding cold Hank’s solution up to 40 ml, digestion ended. The tube was shaken for 1 min, and the suspension was dispensed into a glass container (7.5 cm in diameter and 4.5 cm in height). Cold Hank’s solution was added and aspirated after precipitation. The supernatant was removed (this process was repeated three times). After the last aspiration, the islets were handpicked (Blue Light stereomicroscope, USA), that is, the first picking [17].

### Glucose-stimulated insulin secretion assessment

Glucose-stimulated insulin secretion was assessed at different glucose concentrations (0, 5.6 and 16.7 mM). From the isolated islets of each of the noted animals (6 rats/group, 6 litters per group), two groups of ten islets were picked for each glucose concentration (second picking) and placed in plastic cups (12 cups in total for each condition). All the procedures of islet isolation were carried out on the ice tray. After removal of the excess Hank’s solution, 1 ml of Krebs-Ringer solution (pH = 7.4) [containing in mM: NaCl, 111; KCl, 5; MgCl_2_ 6H_2_O, 1; CaCl_2_, 1; NaHCO_3_, 24 (Merck, Germany); HEPES, 10 (Sigma, USA); and BSA, 0.5 g/dl (Sigma, USA)] containing 0, 5.6 or 16.7 mM of glucose was added to the cups and incubated for 90 min (at the beginning, the cups were gassed with 95% O_2_/5% CO_2_ for 5 min) at 37°C. The supernatant was then removed and stored at -70°C for the insulin assay [17].

### Isolated islets insulin content

At the end of the incubation period and after removing the supernatant of the noted cups, to measure the insulin content of the isolated pancreatic islets, 1 ml of ethanol acid solution (0.18 M HCl in 70% ethanol) was added to the islets left in the cups. The cups were kept at 4°C overnight. On the next day, the solution was centrifuged at 1300 ×g for 10 min and the supernatant was removed to determine its total protein and insulin content [29]. The rat insulin ELISA kit (minimum detection: 0.07 μg/l; Mercodia, Sweden) was used to measure the insulin content and secretion. The measurements were performed in more than one run and the intra and inter assay coefficients of variation were 3.4 and 5.5%.

### The hippocampus insulin content and insulin receptor protein level

After decapitation, the animals’ brain (8 rats/group, 8 litters/group) was placed on ice to remove the hippocampus [4]. Following the Paxinos atlas [30], the brain was divided into two equal parts from the sagittal groove of the left hemisphere on a brain matrix. To prepare the hippocampus, the lysis buffer solution (sodium deoxycholate 0.25% = 0.025 g, NaCl = 0.08 g, SDS = 0.01 g, EDTA = 0.003 g, protease inhibitor cocktail = 1 Tablet, Triton X 100 (0.01%) = 10 λ) was first prepared and added to the hippocampus at a rate of 4 times its weight and homogenized. The suspension was then centrifuged at 3500 ×g for 10 min at 4°C. The supernatant was then separated and transferred into a micro-tube and kept at -70°C. Protein concentrations were determined using the Bradford method [31]. An ultra-sensitive rat insulin ELISA kit (minimum detection: 0.02 μg/l; Mercodia, Sweden) and a rat insulin receptor ELISA kit (minimum detection: 0.2 ng/ml; Zellbio, Germany) were then used to measure the hippocampus insulin content and insulin receptor protein level. The measurements were performed in one run and the intra assay coefficients of variation were 9.2 and 7.1%.

### The correlation of the islets insulin content and secretion and plasma insulin level with the hippocampus insulin content and spatial memory, and the correlation between the hippocampus insulin content and insulin receptor protein level and spatial memory

For assessment of these correlations, the results obtained from the islets insulin content and secretion in response to 5.6 mM of glucose, the fasting plasma insulin concentration, the hippocampus insulin content and insulin receptor protein level and the distance to escape (as an index of spatial memory) were used. The log_10_ of these parameters were calculated and Pearson’s correlation test was used to evaluate the potential relationships between these parameters.

### Statistical analysis

Data were analyzed using SPSS-21 and Graphpad Prism-6 statistical softwares. D’Agostino-Pearson Omnibus normality test was performed and confirmed the normal distribution of all data.

All the data were expressed as mean ± SEM. The Repeated-Measures Analysis of Covariance (ANCOVA) with time or trial taken as a repeated factor and stress as the independent factor and body weight (an important index of nutritional status) as covariate, one-way (with stress taken as factor and body weight as covariate) and two-way (with stress and glucose concentration taken as the factors and body weight as covariate) ANCOVA and Repeated-Measures Analysis of Variance (ANOVA) with time taken as a repeated factor and stress as the independent factor and post hoc Tukey test were performed to compare the offspring variables between the non-STR and STR groups. To use the body weight as covariate, the area under the curve (AUC) of this variable was calculated. Moreover, Pearson’s correlation test was used to assess the relationship between the variables. The unpaired t-test was used to compare the dams’ behavioral parameters and plasma corticosterone concentration between the non-STR and STR groups. Differences less than 0.05 (P<0.05) were considered statistically significant.

## Results

### The effect of maternal separation on the Barnes maze test parameters

**Fig 1:**
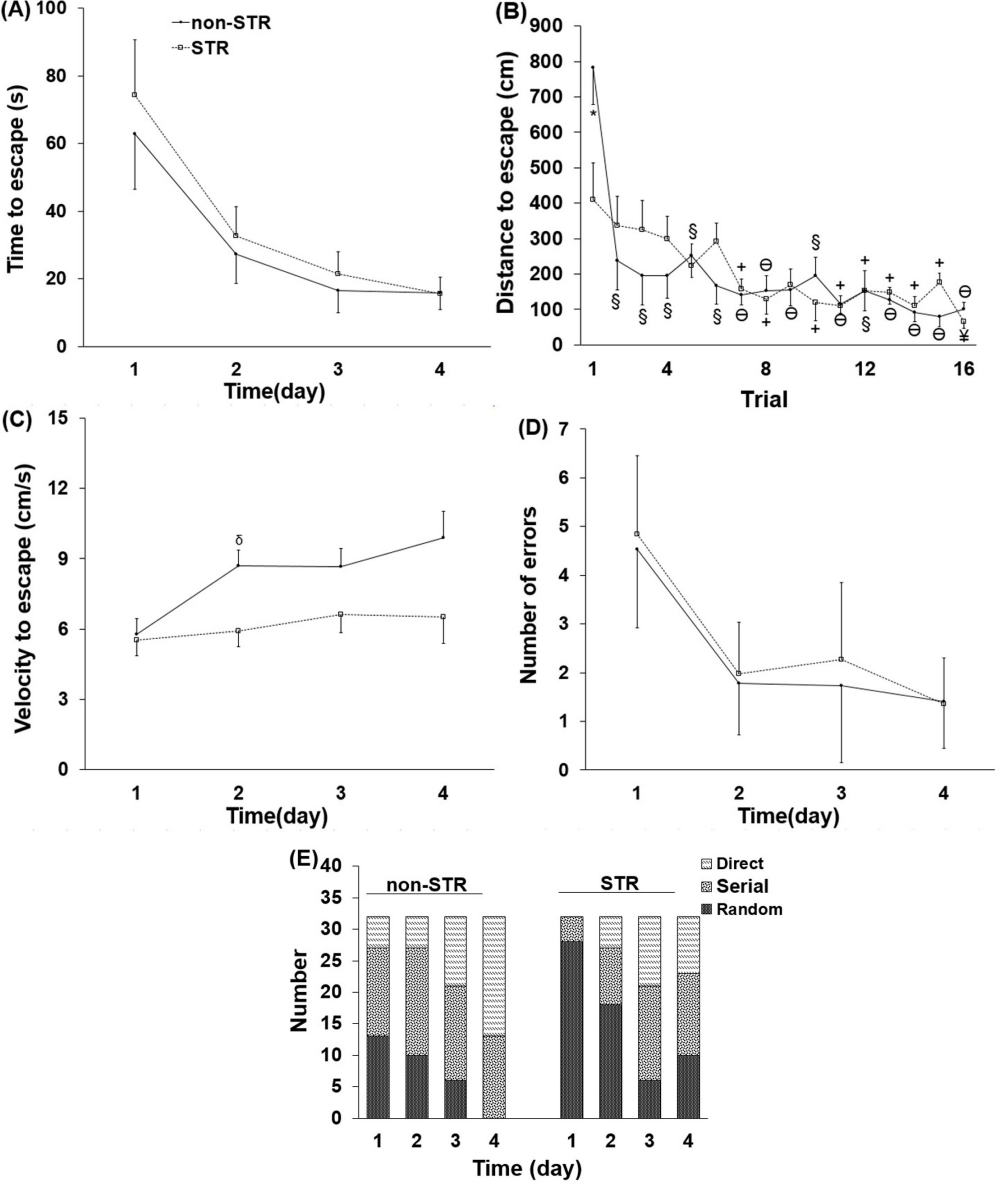
**The effect of maternal separation on time (A), distance (B) and velocity to escape (C), the number of errors (D) and strategy (E) to find the target hole during the 4 days of the memory test (16 trials).** Each point or column represents the mean ± SEM in eight young adult male rats. STR: Stress; Direct: Direct movement from the center of the maze to the escape box; Serial: Serial movement between the holes until reaching the escape box; and Random: Random movement in the center of the maze until reaching the escape box. ^*^P<0.05 vs. non-STR; ^Ɵ^P<0.0001, ^§^P<0.001, ^¥^P<0.01, ^+^P<0.05 vs. trial 1 of the same group; ^δ^P<0.05 vs. day 1 of the same group.

In this study, the time, distance and velocity to escape, and the number of errors to find the target hole as well as the strategy used to reach the escape box were taken as the main parameters measured. Considering body weight as covariate, statistical analysis of these parameters showed that except the distance to find the escape box, the other parameters did not show either a group (Stress) or a trial × group effects; therefore, only the distance to escape is reported trial-to-trial for each group separately. Using body weight as the covariant, ANCOVA showed that the time to escape was not significantly different between the STR and non-STR groups (Fig 1A and S1 Table). The STR group showed a significantly lower distance to escape as compared to the non-STR group only in the first trial (P<0.05; Fig 1B and S2 Table). Further statistical analysis revealed that the distance to escape significantly decreased in trials 2–16 when compared with the first trial in the non-STR group (P<0.001 for trials 2–6, 10 and 12, P<0.0001 for the remaining trials) (Fig 1B and S2 Table). The distance to escape was significantly lower in the STR group in trials 7 and 8 and 10–16 as compared to the first trial (P<0.05 for trials 7 and 8 and 10–15; P<0.01 for trial 16) (Fig 1B and S2 Table). The velocity to escape changed in non-STR group and was significantly higher on day 2 (P<0.05) of the test as compared to the day 1; however, in the STR group, the velocity to escape remained unchanged throughout the experimental days (Fig 1C and S1 Table). The mean errors in finding the escape box (Fig 1D and S1 Table) did not differ significantly between and within the groups.

The animals in the non-STR group showed all three strategies on days 1 to 3 of the memory test, while on day 4, they only demonstrated the direct and serial strategies. On days 1 to 4 of the memory test, the frequency of using the direct strategy increased while the frequency of the random strategy decreased (Fig 1E). In the STR group, the direct strategy was completely absent on day 1 of the test; on days 2–4, although the strategy was present, it did not show an increasing trend. Despite the decrease in the frequency of using the random strategy from days 1 to 4, unlike the non-STR group, this strategy was still present on day 4 of the test (Fig 1E).

### The effect of maternal separation on offspring plasma corticosterone changes

**Fig 2:**
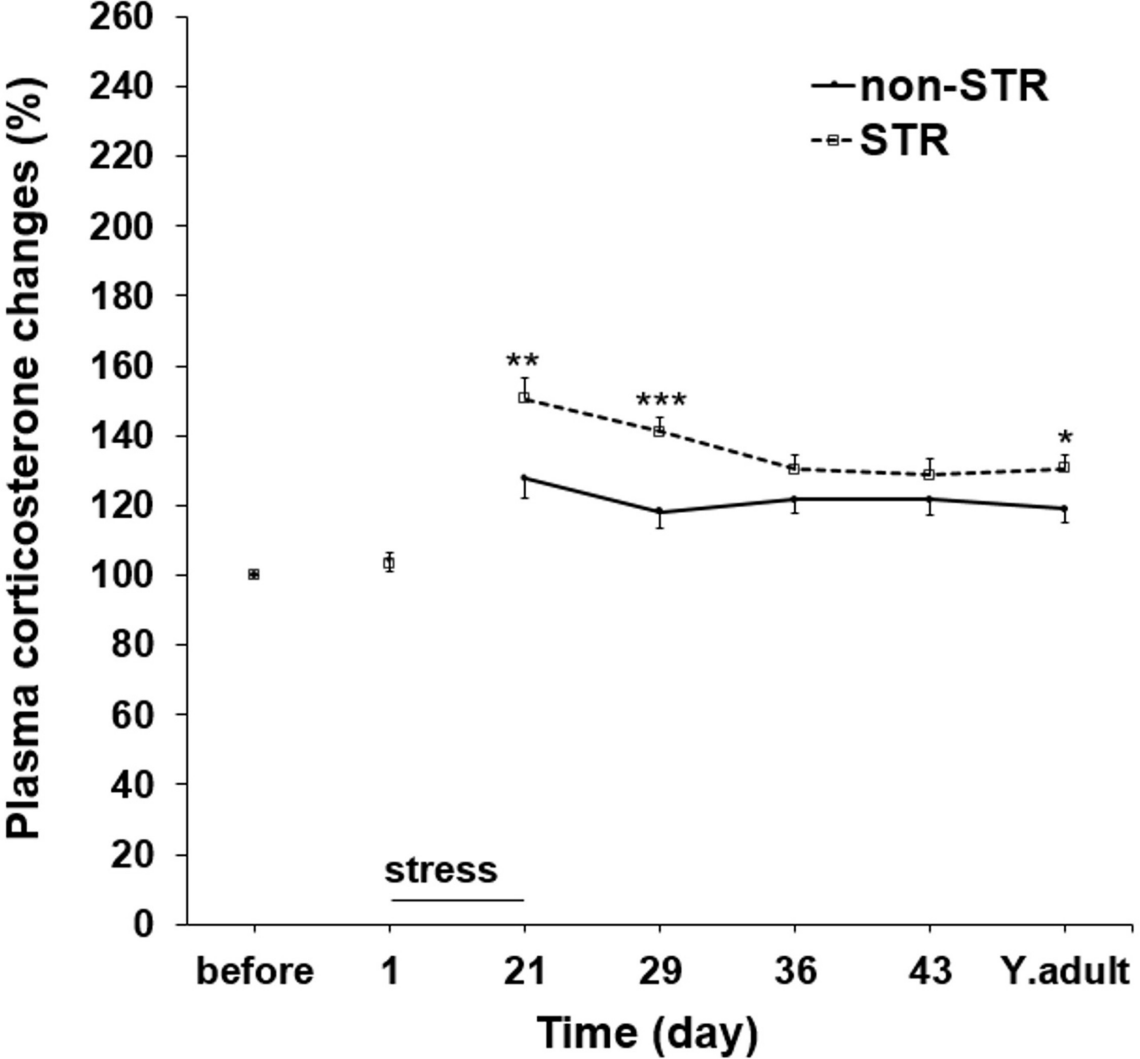
The effect of maternal separation stress on the percentage changes of plasma corticosterone levels. Each point represents the mean ± SEM in ten young adult male rats. STR: Stress; Before: Before stress exposure; Y.adult: young adulthood. ^***^P<0.001, ^**^P<0.01, ^*^P<0.05 vs. non-STR.

The percentage changes of plasma levels of corticosterone were significantly higher in the STR group on PND-21 (P<0.01), PND-29 (P<0.001) and young adulthood (P<0.05) than the non-STR group (Fig 2 and S3 Table).

### The effect of maternal separation on plasma insulin level, islets insulin content and secretion, hippocampus insulin content and amount of insulin receptor protein

In the STR group, maternal separation did not significantly affect plasma insulin levels (S2 Fig). The islets insulin content showed a significant reduction in this group as compared to the non-STR group (P<0.0001) in the presence of 5.6 mM of glucose. No significant differences were observed between the two groups in the presence of 0 and 16.7 mM of glucose (Tables 1 and S4). Moreover, insulin secretion as a percentage of the insulin content did not show any significant difference between the STR and non-STR groups at any glucose concentration (Tables 1 and S4).

**Fig 3:**
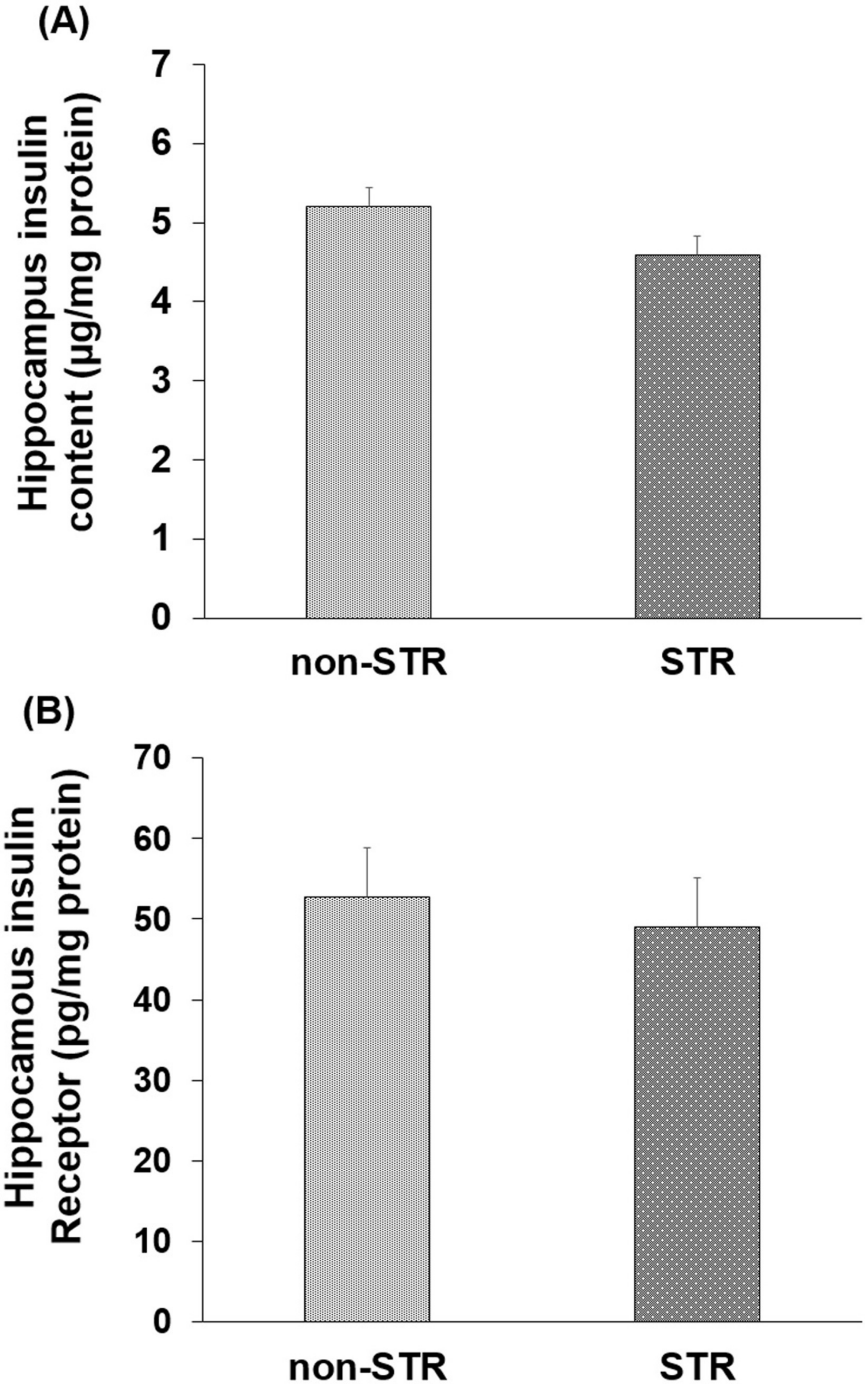
**The effect of maternal separation on the insulin content (A) and insulin receptor protein amount (B) of the hippocampus in young adult male rats.** Each column represents the mean ± SEM in eight young adult male rats. STR: Stress.

The hippocampal insulin content and amount of insulin receptor protein did not show any significant difference between the two groups (Figs 3A and 3B).

**Table 1:** The effect of maternal separation on the isolated pancreatic islets insulin content and insulin secretion as a percentage of insulin content in the presence of different concentrations of glucose in young adult male rats.

	Insulin Content (ng/mg Protein)			Insulin Output/Insulin Content (%)		
	Medium Glucose (mM)			Medium Glucose (mM)		
Group	0	5.6	16.7	0	5.6	16.7
Non-STR	23.65±9.41	92.05±9.41	105.88±9.41	71.31±5.14	83.71±5.14	92.67±5.14
STR	23.06±9.41	14.50 ±9.41^****^	88.61 ±9.41	68.91±5.14	71.17±5.14	92.68±5.14

Values are expressed as the mean ± SEM in six young adult male rats (two groups of ten islets for each concentration of glucose in each animal); STR: Stress,

^****^P<0.0001 vs. non-STR of the same glucose concentration.

### Correlation of the islets insulin content and secretion and plasma insulin level with the hippocampus insulin content and spatial memory, and correlation between the hippocampus insulin content and insulin receptor protein level and spatial memory

The islets insulin content and secretion and plasma insulin level did not correlate significantly with the hippocampus insulin content and spatial memory. Moreover, there were no statistical correlations between the hippocampus insulin content and insulin receptor protein level and spatial memory (Table 2), whereas, in the STR group, a positive corelation between hipocampus insulin content and insulin receptor protein amount was observed (Table 2).

**Table 2:** Correlation of the islets insulin content and secretion and plasma insulin level with the hippocampus insulin content and spatial memory, and correlation between the hippocampus insulin content and insulin receptor protein level and spatial memory.

Variable	Hippocampus insulin content		Spatial memory	
	Pearson’s Coefficient	P-Value	Pearson’s Coefficient	P-Value
**Non-STR Group**				
Insulin content of the islets (5.6)	0.05	0.92	0.24	0.65
Insulin secretion from the islets (5.6)	0.17	0.74	0.19	0.72
Plasma insulin concentration	0.50	0.30	0.57	0.24
Hippocampus insulin content	-	-	-0.09	0.86
Hippocampus insulin receptor protein level	0.58	0.22	0.51	0.31
**STR Group**				
Insulin content of the islets (5.6)	0.16	0.75	0.10	0.89
Insulin secretion from the islets (5.6)	0.34	0.49	0.15	0.77
Plasma insulin concentration	- 0.48	0.32	0.34	0.50
Hippocampus insulin content	-	-	0.02	0.96
Hippocampus insulin receptor protein level	0.82	0.05	0.43	0.39

### The effect of maternal separation stress on the dams’ behavior and plasma corticosterone level

**Fig 4:**
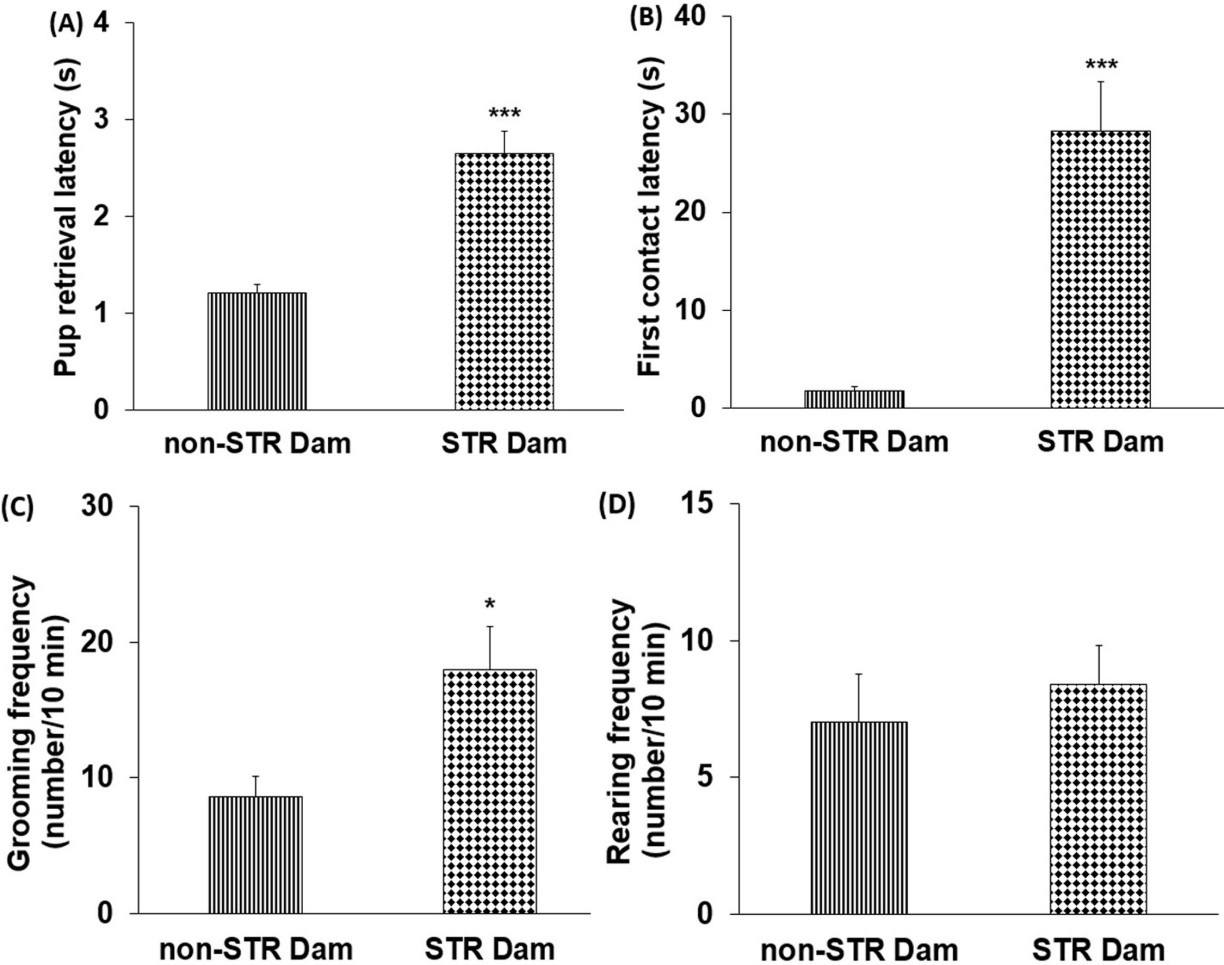
**The effect of maternal separation on the pup retrieval latency (A), first contact latency (B), grooming (C) and rearing frequencies (D) in non-STR and STR dam groups.** Each column represents the mean ± SEM in six female rats. STR: stress. *P<0.05, ***P<0.001 vs. non-STR Dam.

After the pup retrieval test, both groups of dams returned all the pups to the nest during the cut-off time, but the STR dams returned the pups later than the non-STR dams (P<0.001) (Fig 4A). The time spent to touch or sniff the first pup was significantly longer in the STR dams as compared to the non-STR dams (P<0.001) (Fig 4B). Moreover, the grooming frequency was significantly higher in the STR dams than the non-STR dams (P<0.05) (Fig 4C), while the rearing frequency showed no significant difference (Fig 4D).

**Fig 5:**
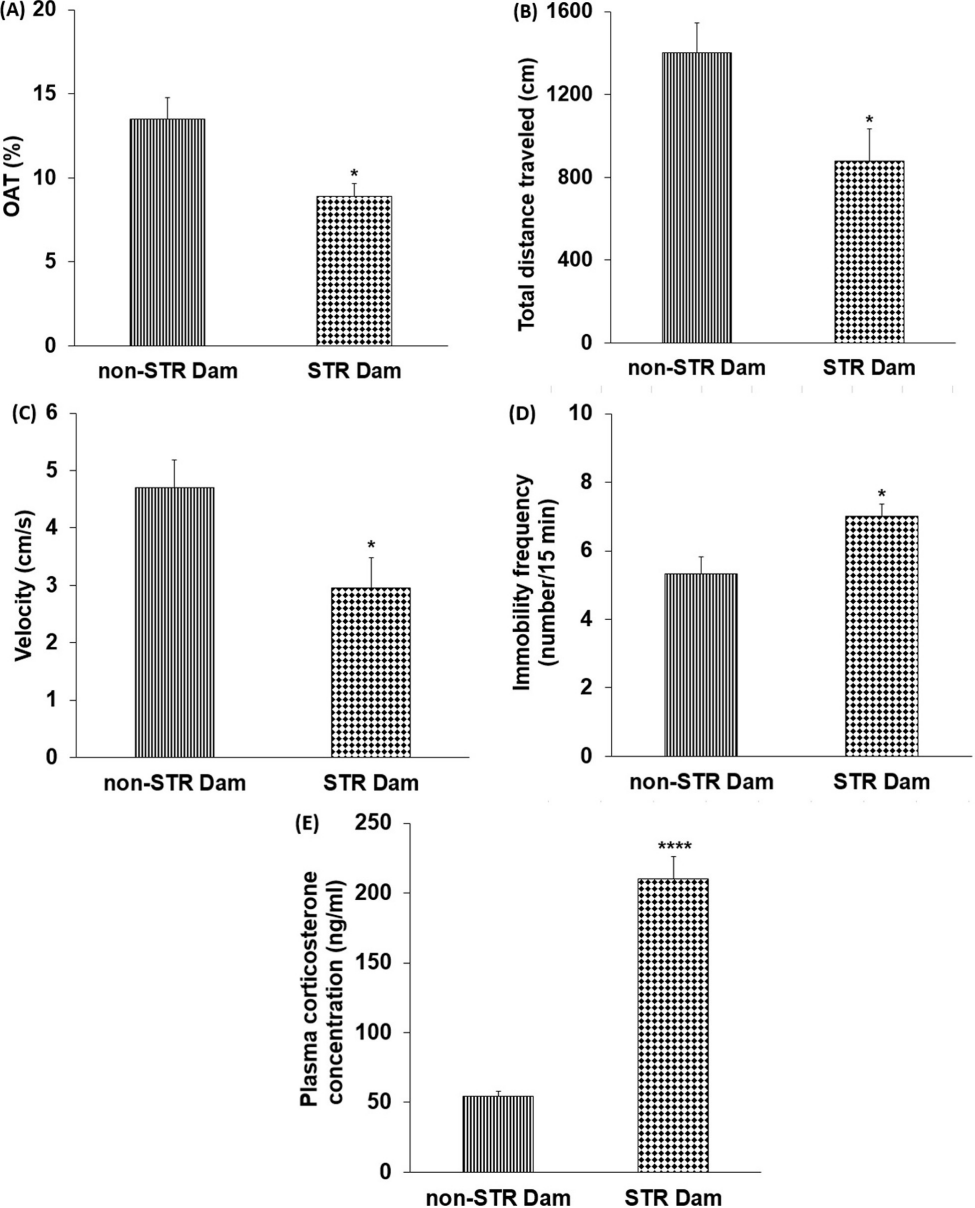
**The effect of maternal separation on the percentage of open arm time (OAT %) (A), total distance traveled (B) and velocity of movement (C) in elevated plus maze, frequency of immobilization (D) during forced swim test, and plasma corticosterone level (E) in non-STR and STR dam groups.** Each column represents the mean ± SEM in six female rats. STR: stress. ^P^*P<0.05, ****P<0.0001 vs. non-STR Dam.

The EPM study showed that OAT% was significantly lower in the STR dams than the non-STR dams (P<0.05) (Fig 5A). In addition, the total distance traveled by the STR dams as well as the velocity of movement were significantly lower than the non-STR dams (P<0.05) (Fig 5B and 5C, respectively). Moreover, the results of the forced swimming test showed a higher frequency of immobility in the STR dams than the non-STR dams (P<0.05) (Fig 5D).

The data on plasma corticosterone levels revealed that this hormone was significantly higher in the STR dams as compared to the non-STR dams (P<0.0001) (Fig 5E).

### The body weight and length of the pups during the weaning time

**Fig 6:**
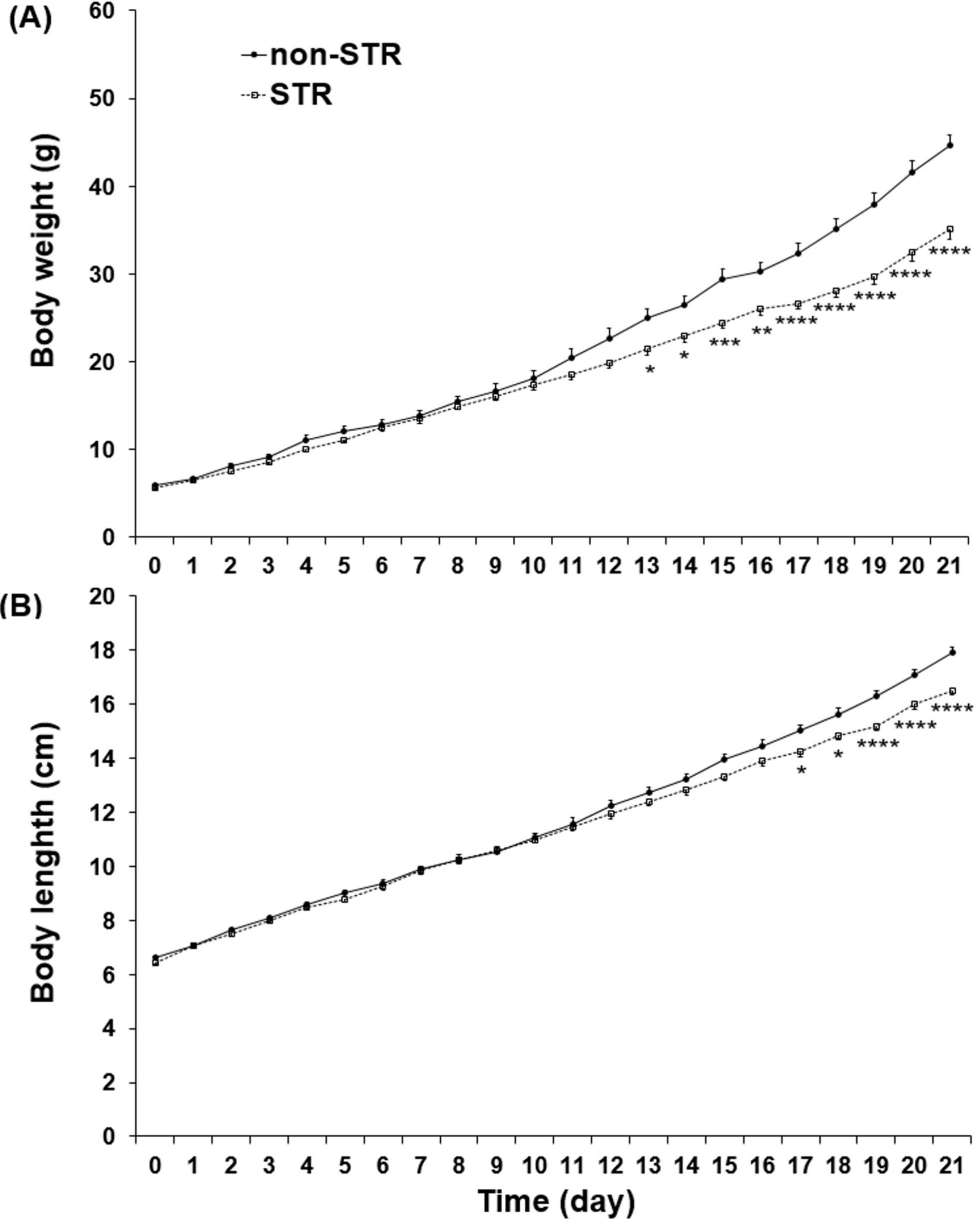
**The effect of maternal separation on the pups’ body weight (A) and length (B) in the non-STR and STR groups during the weaning time.** Each point represents the mean ± SEM in 18 male rats. STR: stress. *P<0.05, **P<0.01, ***P<0.001, ****P<0.0001 vs. non-STR.

The results indicated that the body weight was significantly lower in the STR pups than the non-STR pups from PND-13 to PND-21 (PND-13 and PND-14, P<0.05; PND-15, P<0.001; PND- 16 P<0.01 and PND- 17 to PND-21, P<0.0001) (Fig 6A and S5 Table). The head to tail length + tail length measurements indicated that the body length was significantly lower in the STR pups than the non-STR pups from PND-17 to PND-21 (PND-17 and PND- 18, P<0.05; PND-19 to PND- 21, P<0.0001) (Fig 6B and S5 Table).

## Discussion

In the present study, maternal separation stress interfered with memory formation. The time to escape, which is considered as the main parameter in the Barnes maze, did not show any significant difference between the STR and non-STR groups; however, the distance to escape was another important parameter that showed a steep reduction in the non-STR group as compared to the STR group after the first trial. The distance to escape, which showed a trial-dependent deficit in performance on the Barnes maze induced by maternal separation, could be a sign of abnormal development of the hippocampus as one of the main sites involved in spatial memory in the brain [3, 13]. Several reports confirm that maternal separation results in long-term changes in the morphology of the cognitive brain structures, such as a reduction in the hippocampal volume and size of the pyramidal and granular layers [32], a reduction in the number of neurons in the dentate gyrus [33] and a reduction in the CA1 pyramidal cell layer and CA3 area and volume [8], which could lead to memory impairment [34, 35]. Figueiredo et al. [8] showed that pups have a slower linear growth and poorer weight gain following prolonged maternal separation as compared to the controls, which could be a characteristic of under-nutrition and could also be the cause of changes in the pups’ hippocampus morphology and function [36]. In contrast to the STR group, assessment of the strategy used to reach the escape box in the non-STR group revealed an increase in the frequency of using the direct strategy and a reduction in the use of the random strategy over time. Studies have shown that the prefrontal cortex is the main part of the brain involved in strategy determination by the rats in different maze tasks [37–39]. Considering the effects of stress hormones on the reduction of neuronal structures in the medial prefrontal cortex [40, 41], it could be concluded that the use of an abnormal strategy in the STR group may be the result of the interaction between stress hormones and neurons in the prefrontal cortex in these animals. To clarify this hypothesis, further experiments including morphological and electrophysiological studies in this brain region are needed. In addition, as strange as it is that the time to escape was not different between the groups (as change in this specific parameter is an extensively reported effect of the ELS [42–44] and thus is expected), it is possible that testing was more sensitive to strategy than to latency index. Another sign of the interference of maternal separation stress in memory formation is that, in contrast to the non-STR group, the rats in the STR group showed no significant increase in their velocity to escape as compared to the first day when they were tested using the Barnes maze. One explanation is that the STR group had a degree of stress-induced anxiety [45–47], and although these animals were pre-exposed to the maze, they were not habituated to its environment.

The pups’ body weight measurement in the present study showed that, in line with the aforementioned study [8], the STR group had a poor weight gain from PND-13 to PND-21. The under-nutrition might therefore be considered as one of the potential causes of the blunted memory formation observed in the present study [8]. Nonetheless, other studies conducted using a maternal separation protocol partly similar to the present one have not shown any significant deficits in body weight gain in pups separated from their mothers early in life [48, 49]. One should not rule out the role of plasma corticosterone elevation in the STR rats, which might cause hippocampal structural changes leading to impaired learning [50, 51]. In agreement with this hypothesis, previous studies have shown that the long-term administration of corticosterone in rodents leads to the damage of the hippocampal neurons and increases the time and distance to escape and the number of errors in the Barnes maze paradigm [25, 52].

In this study, on PND-1, no significant difference was observed between the groups in relation to plasma corticosterone levels, which may be due to the hypo-responsiveness of the HPA axis in the postnatal period [53]. The elevated basal plasma corticosterone concentration, especially in young adulthood, may be a sign of the persistent effects of maternal separation stress and/or under-nutrition on HPA axis programming [7, 54], which might be manifested as a reduction in glucocorticoid receptors in the hippocampus and/or their malfunction in the other brain regions [55, 56], the increased release of corticotropin-releasing factor or CRF [57], the adrenocorticotropic hormone or ACTH [58] and/or the altered responsiveness of the adrenal glands in ELS-exposed rats [59]. Maybe, these mechanisms are also involved in the changes in plasma corticosterone following maternal separation in this study. The fact that these parameters were not assessed may be a limitation of this study. Koe et al. [60] reported that applying this kind of stress from PND-2 to PND-21 in male Wistar rats does not change the basal plasma corticosterone concentration on PND-56 as compared to the controls. Further studies might clear these discrepancies. In line with the results of some previous studies [61–63], plasma insulin concentrations did not show significant difference between the STR and non-STR groups in this study. Meanwhile, Solas et al. [4] reported that maternal separation from PND-2 to PND-21 reduces plasma insulin levels in male Wistar rats at three and 18 months of age. This disparity in results could be due to the different experimental protocols used.

The present findings showed a reduction in the islets insulin content in response to 5.6 mM of glucose in the STR group as compared to the non-STR group. Few studies have focused on the effect of ELS on insulin content and secretion from isolated islets [63]. This reduction could reflect a change in the islets glucose-sensing machinery and their sensitivity to glucose due to changes in glucose transporter 2 (GLUT2) expression, glucokinase activity and the potassium and calcium channels’ activity [64], likely due to the altered pancreatic islets programming as a result of the persistant increase in plasma corticosterone concentrations in the STR group, especially during infancy [65]. In the STR group, the results of plasma insulin concentration and the isolated islets insulin content in response to basal concentration of glucose (5.6 mM) are not consistent, which might be explained by possible change in programming of hepatic insulin degrading enzyme expression level due to maternal separation and/or under-nutrition [66]. However, this hypothesis should be assessed in future studies.

In the present study, the hippocampus insulin content and insulin receptor protein amount did not significantly change in the STR group as compared to the non-STR group. Reports on the effects of ELS on hippocampal insulin content are limited; nonetheless, in line with the present findings, maternal separation (3 h/day) from PND-2 to PND-21 led to a non-significant reduction of insulin receptor expression and its phosphorylated levels in Wistar rats at three months of age [4]. In contrast, the same period of maternal separation led to a significant reduction in the hippocampus amount of insulin receptor protein in male Wistar rats at 60–75 days of age, which might have been caused by the epigenetic changes in the hippocampus [67]. One study reported that Streptozotocin-treated rats with low plasma insulin content have impaired hippocampal neurogenesis, synaptic plasticity and learning, and thus demonstrated the role of reduced hippocampus insulin in memory performance [68]. Moreover, intrahippocampal insulin administration improved spatial learning and memory in the control rodents [64]. Many other studies have also shown that insulin receptors and content in the hippocampus are involved in memory processing by disabling the proapoptotic mechanisms and/or acting as a growth factor or triggering the release of the neurotrophic factors to support neuronal survival [4, 69] and the induction of long-term potentiation (LTP) and long-term depression (LTD), which are the basic processes in learning and memory [4]. Insulin may also play a role in the regulation of the neurotransmitters that are involved in memory, such as norepinephrine and acetylcholine [70]. Insulin also showed an anti-stress function, as intranasal insulin attenuated cortisol secretion and HPA axis response to stress [69]. The statistical analysis of the present study, however, did not show any significant change in hippocampal insulin content and insulin receptor protein level and any correlation between these parameters and spatial memory. According to these findings one may conclude that the spatial memory formation is independent of hippocampal insulin content and insulin receptor protein level in the STR group.

In this study, the anxiety and depressive-like behavior of the dams were also evaluated. Previous studies have shown that maternal separation can affect dams’ behavior (that is, it may induce anxiety and depression-like behavior) and increase active maternal care [9, 71], which might in turn alter the offspring’s behavior. The present findings are in agreement with the results of these studies, as the STR dams showed anxiety and depressive-like behaviors indicated by a lower OAT% and locomotor activity in the EPM and a higher immobility frequency in the forced swimming test. Moreover, they showed less nursing behaviors during the retrieval test. As expected, plasma corticosterone levels were also high in these animals, and previous studies have also revealed a direct relationship between anxiety and depression, and plasma corticosterone levels [72]. The dams’ anxiety and depression during the weaning period may interfere with the pups brain development and body growth in some ways; first, they reduced nursing behavior and the milk offered to the pups (because of the effects of the neurohormones secreted in the hypothalamic PVN nucleus); second, several stress-related neurohormones and hormones may be secreted into the dams’ milk and can reach the pups and in turn affect their growth and interfere with the programming of multiple developing systems, including the neuroendocrine and metabolic systems [73]. Overall, all these mechanisms can explain the present findings.

Previous studies have shown that the brain and hippocampus insulin content depend on the plasma insulin concentration transferred to the cerebrospinal fluid (CSF) through the insulin receptors in the blood-brain-barrier (BBB) and insulin production in the brain [74]. However, in the present study, the islets insulin content and secretion, and the insulin content of the hippocampus were not statistically correlated; also, the plasma insulin concentration and the hippocampus insulin content did not differ between the STR and non-STR groups. These findings suggest that the blunted spatial memory observed in the STR group could be independent of peripheral and hippocampal insulin content.

## Conclusion

According to the results of the present study, unexpectedly, the time to escape as an important index in Barnes maze paradigm was not affected by maternal separation stress and showed no significant change in offspring of the STR group. However, it appears that the main behavioral effect of the maternal separation stress in the spatial memory task was impairment of the strategy used by the animals to reach the escape box which may indicate that testing was more sensitive to strategy than to the latency index. This may also indicate that maternal separation stress affects brain regions other than the hippocampus. In addition, due to the fact that the maternal separation procedure significantly affected maternal behavior, so much so that offspring belonging to the STR group had reduced body weight and length as compared to offspring of the non-STR group, it could be suggested that the offspring were undernourished. Therefore, alterations in the islets insulin content in the pups are not the only consequences of maternal separation that could explain the behavioral results of offspring. In conclusion, it should be further considered that both maternal separation and early life malnutrition have been directly (and mechanistically) linked to alterations in cognitive processes, such as learning and memory, later in life in ways that are not dependent on peripheral and hippocampal insulin content.

## Supporting information

S1 Fig The LED light spectrum. The LED light had a visible spectrum range from about 440 to 700 nm with a peak at lambda = 590 nm.
(TIF)

S2 Fig Maternal separation effect on plasma insulin concentration in young adult male rats. Each column represents mean ± SEM of 14 young adult male rats. STR: stress.
(TIF)

S1 Table The F values of the repeated measures ANCOVA of time and velocity to escape and the number of errors to find the target hole during the 4 days of the memory test (16 trials). (DOCX)

S2 Table The F values of the repeated measures ANCOVA of distance to escape to find the target hole during the 4 days of the memory test (16 trials). (DOCX)

S3 Table The F values of the repeated measures ANCOVA of plasma corticosterone levels in young adult male rats. (DOCX)

S4 Table The F values of the two-way ANCOVA of the isolated islets insulin content and insulin output/insulin content in the presence of different glucose concentrations in young adult male rats. (DOCX)

S5 Table The F values of the repeated measures ANOVA of the pups’ body weight and length in the non-STR and STR groups during the weaning time. (DOCX)
