# Correction: Maternal separation blunted spatial memory formation independent of peripheral and hippocampal insulin content in young adult male rats

**DOI:** 10.1371/journal.pone.0210893

**Published:** 2019-01-10

**Authors:** Soheila Maghami, Homeira Zardooz, Fariba Khodagholi, Fatemeh Binayi, Roya Ranjbar Saber, Mehdi Hedayati, Hedayat Sahraei, Mohammad Ali Ansari

There is an error in affiliation 5 for author Mehdi Hedayati. Affiliation 5 should be: Cellular and Molecular Endocrine Research Center, Research Institute for Endocrine Sciences, Shahid Beheshti University of Medical Sciences, Tehran, Iran [1].
