# Supplementary figures and images for: Maternal separation blunted spatial memory formation independent of peripheral and hippocampal insulin content in young adult male rats
Source: PLoS One. 2018 Oct 17;13(10):e0204731. doi: 10.1371/journal.pone.0204731 (PMC6192583; doi:10.1371/journal.pone.0204731)

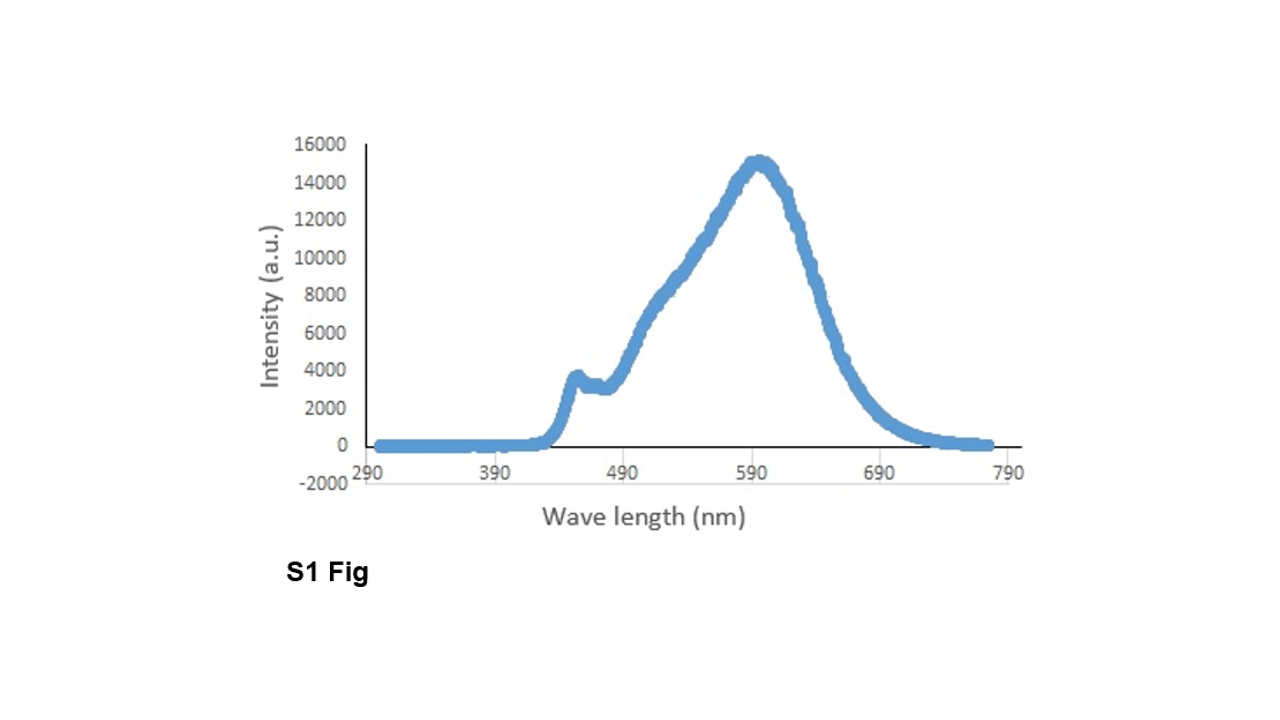

Supplement: S1 Fig — The LED light had a visible spectrum range from about 440 to 700 nm with a peak at lambda = 590 nm. (TIF) [file pone.0204731.s001.TIF]

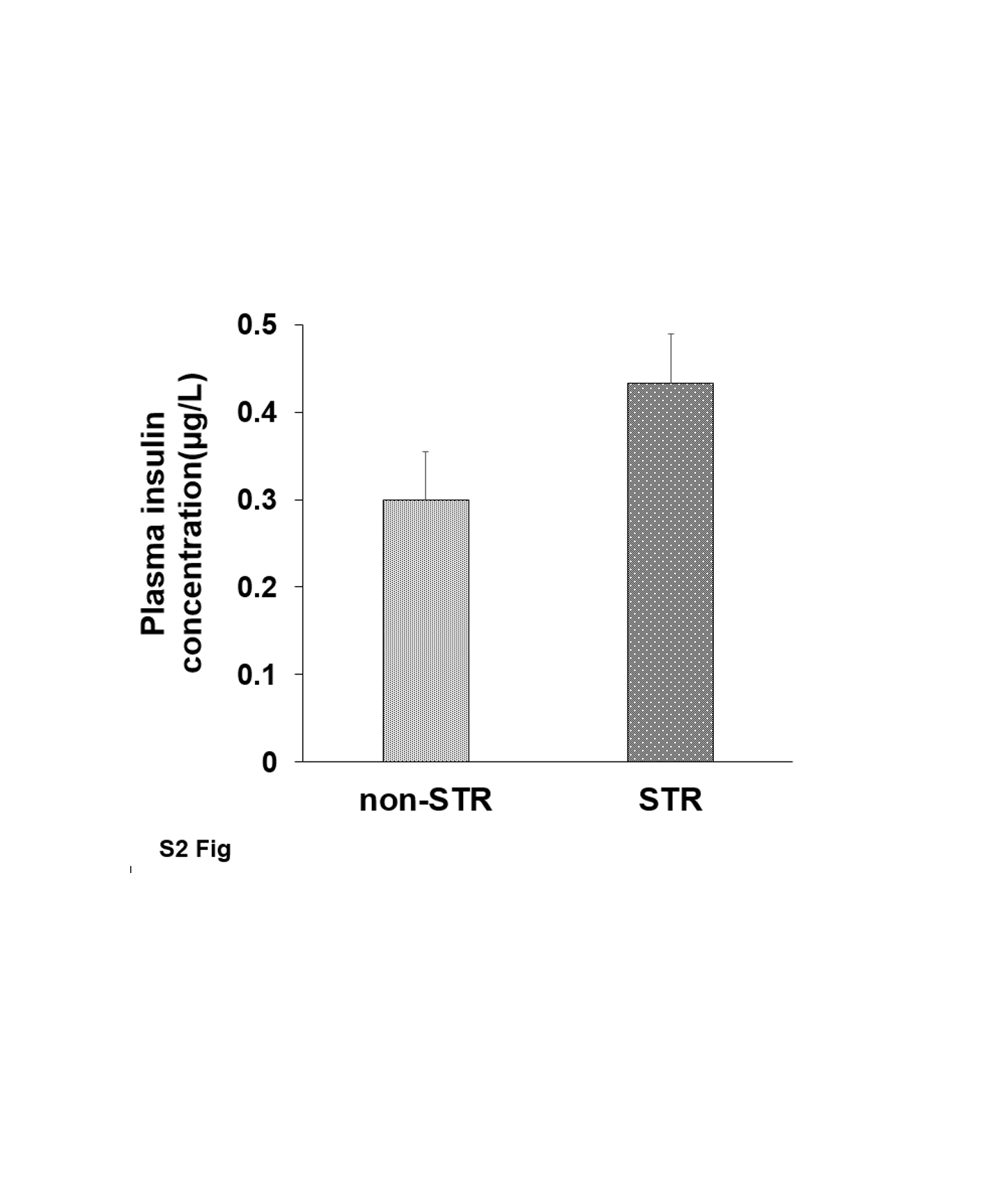

Supplement: S2 Fig — Each column represents mean ± SEM of 14 young adult male rats. STR: stress. (TIF) [file pone.0204731.s002.TIF]
