# Supplementary material for: Maternal separation blunted spatial memory formation independent of peripheral and hippocampal insulin content in young adult male rats
Source: PLoS One. 2018 Oct 17;13(10):e0204731. doi: 10.1371/journal.pone.0204731 (PMC6192583; doi:10.1371/journal.pone.0204731)
Supplement: S1 Table — (DOCX) [file pone.0204731.s003.docx]

**S1 Table.**

| Factors | Time (day) | Stress | Time (day) * Stress |
| --- | --- | --- | --- |
| Time to escape | F(3, 42)=0.356  P=0.785 | F(1, 14)=0.228  P=0.641 | F(3, 42)=0.183  P=0.908 |
| Velocity to escape | F(3, 42)=0.728  P=0.541 | F(1, 14)=4.080  P=0.064 | F(3, 42)=2.756  P=0.055 |
| Number of errors | F(3, 42)=2.017  P=0.127 | F(1, 14)=0.025  P=0.877 | F(3, 42)=0.026  P=0.994 |
