# Supplementary material for: Maternal separation blunted spatial memory formation independent of peripheral and hippocampal insulin content in young adult male rats
Source: PLoS One. 2018 Oct 17;13(10):e0204731. doi: 10.1371/journal.pone.0204731 (PMC6192583; doi:10.1371/journal.pone.0204731)
Supplement: S2 Table — (DOCX) [file pone.0204731.s004.docx]

**S2 Table.**

| Factor | Trial | Stress | Trial * Stress |
| --- | --- | --- | --- |
| Distance to escape | F(15, 210)=1.835  P=0.032 | F(1, 14)=0.023  P=0.881 | F(15, 210)=2.129  P=0.010 |
