# Supplementary material for: Maternal separation blunted spatial memory formation independent of peripheral and hippocampal insulin content in young adult male rats
Source: PLoS One. 2018 Oct 17;13(10):e0204731. doi: 10.1371/journal.pone.0204731 (PMC6192583; doi:10.1371/journal.pone.0204731)
Supplement: S3 Table — (DOCX) [file pone.0204731.s005.docx]

**S3 Table.**

| Factor | Time (day) | Stress | Time (day) * Stress |
| --- | --- | --- | --- |
| Plasma corticosterone level | F(5, 90)=0.289  P=0.918 | F(1, 18)=5.171  P=0.036 | F(5, 90)=3.131  P=0.012 |
