# Supplementary material for: Maternal separation blunted spatial memory formation independent of peripheral and hippocampal insulin content in young adult male rats
Source: PLoS One. 2018 Oct 17;13(10):e0204731. doi: 10.1371/journal.pone.0204731 (PMC6192583; doi:10.1371/journal.pone.0204731)
Supplement: S4 Table — (DOCX) [file pone.0204731.s006.docx]

**S4 Table.**

| Factors | Glucose | Stress | Glucose * Stress |
| --- | --- | --- | --- |
| Insulin content | F(2, 66)=33.74  P<0.0001 | F(1, 66)=12.447  P<0.001 | F(2, 66)=10.009  P<0.0001 |
| Insulin Output/Insulin content | F(2, 66)=10.844  P<0.0001 | F(1, 66)=1.021  P=0.316 | F(2, 66)=0.907  P=0.409 |
