# Supplementary material for: Maternal separation blunted spatial memory formation independent of peripheral and hippocampal insulin content in young adult male rats
Source: PLoS One. 2018 Oct 17;13(10):e0204731. doi: 10.1371/journal.pone.0204731 (PMC6192583; doi:10.1371/journal.pone.0204731)
Supplement: S5 Table — (DOCX) [file pone.0204731.s007.docx]

**S5 Table.**

| Factors | Time (day) | Stress | Time (day) * Stress |
| --- | --- | --- | --- |
| Body weight | F(21, 714)=661.00  P<0.0001 | F(1, 34)=16.63  P=0.0003 | F(21, 714)=15.59  P<0.0001 |
| Body length | F(21, 714)=1414  P<0.0001 | F(1, 34)=5.961  P=0.0200 | F(21, 714)=5.946  P<0.0001 |
